# Crafting British medicine in the Empire: the establishment of medical schools in India and Canada, 1763–1837

**DOI:** 10.1017/mdh.2024.6

**Published:** 2024-04

**Authors:** Martin Robert

**Affiliations:** Banting Postdoctoral Fellow, Centre for History in Public Health, London School of Hygiene and Tropical Medicine, 15-17 Tavistock Place, London, WC1H 9SH, United Kingdom

**Keywords:** Medical schools, British Empire, India, Canada, Professionalisation, Nineteenth century

## Abstract

In the early nineteenth century, medical schools became a growing means of regulating medicine in the British Empire, both in the metropole and in two colonies: India and Canada. By examining the establishment of medical schools in Calcutta, Bombay, Madras, Quebec City, Montreal and Toronto between the end of the Seven Years’ War and the beginning of the Victorian era, this article argues that the rise of the British Empire was a key factor in the gradual replacement of private medical apprenticeships with institutional medical education. Although the imperial state did not implement a uniform medical policy across the British Empire, the medical schools established under its jurisdiction were instrumental in devising a curriculum that emphasised human dissection, bedside training in hospitals and organic chemistry as criteria of medical competence.

At the turn of the nineteenth century, medical schools became a growing means of regulating medicine both in the metropole as well as in two colonies of the British Empire: India and Canada.^1^ Before those in India and Canada, the only colonial medical schools established in the British Empire were in Pennsylvania (1765) and New York (1767). However, both severed their official links with the imperial state and were transformed after the American Revolutionary War (1775–1783), as discussed by Christopher D. E. Willoughby in this issue.^2^ Medical schools were not yet the main route for training doctors. It was a century or so later that they began to appear in most countries of the world, following a significant trend towards the replacement of private medical apprenticeships with institutional medical education. The terms “India” and “Canada” are used here for the sake of convenience, to roughly locate the parts of the world under discussion using today’s terminology. However, the political geography of these territories was in constant flux at the time, particularly in North America. For this reason, the geographical focus of this article is more on the cities under British jurisdiction where medical schools were established.

Although the medical schools founded in the early nineteenth century in Calcutta, Bombay, Madras (now Kolkata, Mumbai, Chennai), Quebec City, Montreal and Toronto were not the result of a uniform policy imposed by the British imperial capital, their curricula were largely based on similar principles: they promoted training in anatomical dissection, bedside teaching and organic chemistry as criteria for distinguishing competent practitioners from quacks. It was clear to their founders that these schools had to be associated with dissecting rooms and teaching hospitals and that they should include courses in materia medica, chemistry and botany. This pedagogy created an *esprit de corps* among the medical representatives of the empire that would later facilitate the regulation of the profession through medical school admissions, diplomas and licensing.^3^ In their efforts to create some sort of core medical training – although there were significant variations between schools and territories – doctors and surgeons of the empire sought to draw new distinctions between their work and pre-colonial health practises in the colonies, as well as to regulate medical practice more strictly than previous generations of health practitioners.

The historiography on medicine in the British Empire has rarely included South Asia and North America in the same analysis.^4^ This article does so by linking the well-documented history of medical education in India with the comparatively less studied one in Canada for the early nineteenth century. It seeks to explain why medical education took the form of medical schools in these two British colonies but not in others at the time. By way of comparison, the first British medical school in Australia was founded in 1856, while the first in the Caribbean was founded in 1948.^5^

The period covered is from the end of the Seven Years’ War (1756–1763) to the beginning of the Victorian era (1837). After the Seven Years’ War, France ceded Canada to the British Crown, while large parts of India were incorporated into the British Empire. Soon after, the American Revolutionary War was a major setback for the British government, which turned its attention to other parts of the world, particularly India.^6^ The wars of the French Revolution (1792–1802), the Napoleonic Wars (1803–1815) and local conflicts, such as the First Anglo-Burmese War (1824–1826), also had territorial implications in the British colonies. In their wake, the establishment of medical schools in both India and Canada was supported by interrelated political, scientific, military, religious and commercial ambitions. British colonial territories in North America and South Asia were not yet strictly categorised as non-settler colonies (with climates considered hostile to colonisation) or settler colonies (with climates considered favourable to settlement), a distinction that would structure the British Empire later in the nineteenth century.^7^ Their respective administrations both reported to the same imperial capital.

The fact that there were common disciplines in medical schools in different parts of the British Empire does not mean that this was the beginning of a linear and unanimous process of standardisation of medical education. At the time, medical education was far from uniform, including in Britain, where it would not become much more so until well after the passing of the Medical Act of 1858. What this article seeks to show is the beginning of a long and tortuous process by which certain practices and knowledge presented as core to medical education, such as anatomical dissection, bedside teaching and organic chemistry, began to be imposed, contested and negotiated under the same imperial jurisdiction on a scale that spanned the globe, long before the advent of international health organisations. It shows that in the early nineteenth century, the conflicting impulses to impose norms by suppressing local specificities and to allow specificities around core competence criteria coexisted. The first British medical schools in India are a case in point: they initially integrated to some extent the pre-colonial Indian medical traditions of Ayurveda and Unani before being abolished after a few years and replaced by institutions that did not. This complex, ongoing struggle over what is non-negotiable in order to become a recognised doctor and what can vary according to circumstances continues to create tensions at all levels today, including internationally, through conflicts over the recognition of medical qualifications between countries. It is, therefore, relevant to trace the long history of these tensions and how they have changed in territories that have been under colonial rule.

## British medical education in India (1763–1835)

British medical education in India was institutionalised top-down by local imperial administrations, mainly to serve British East India Company (BEIC) troops and staff. As sea voyages were long, expensive and unreliable, the aim was to reduce dependence on doctors from Britain. Training in British medical institutions in India was influenced by concerns about the effects of ‘hot climates’ on European bodies. For some years, it was also shaped by British Orientalists who sought to combine Indian and British health practises and knowledge, including in the preparation of remedies. British colonial hospitals in India relied heavily on local bazaars for their supply of medicines.^8^ Overall, because of the convergence of military and economic interests and a certain reverence that some of the British had for what they conceived of as Indian civilisation, the imperial administration invested more in its medical schools in India than in their Canadian counterparts.

By the end of the Seven Years’ War, the British presence in India consisted of little more than trading posts and military bases run by the BEIC, whose medical services employed a growing number of health practitioners.^9^ The hospital established at Fort St. George in Madras had been caring for the health of European soldiers, sailors and staff since 1664, and many European-trained surgeons had come to India to work for the BEIC since the seventeenth century.^10^ They largely looked after the company’s employees and participated in public projects such as the hospitals for the poor in Calcutta.^11^ Some, such as the surgeon Busick Harwood, benefited from their time in South Asia by looking after the health of Indian princes.^12^

The medical environment into which these practitioners entered was structured by institutionally transmitted Indian health care traditions. Unani was taught in Islamic madrasas, seminaries or courts of the Indian aristocracy.^13^ The Ayurvedic tradition was taught in private settings by mentors.^14^ Occasionally, colonial administrators learned about diseases and remedies from these Indian medical circles. For example, in 1817, during a cholera epidemic in Bengal, the BEIC Medical Boards sought support and recommendations from Indian practitioners.^15^ As Mark Harrison notes, it is also likely that the surgeons James Wilson and Gilbert Pasley learned from Indian practitioners in Madras in the 1790s about the use of mercury to treat various diseases. Echoes of such experiments in the colonies were sometimes heard elsewhere in the empire, as when surgeon Helenus Scott used nitric acid to treat syphilis in Bombay, which was replicated in other British colonies and in Britain.^16^ The use of Indian remedies by imperial doctors and surgeons followed a similar logic to that of British medical education in the colonies: locally produced remedies were seen as better suited to local contexts, more readily available and less expensive than those coming from Britain.

The medical training sponsored by the BEIC developed in this context as an adjunct to the company’s commercial and military activities. Projit Mukharji notes that towards the end of the eighteenth century, the BEIC employed about a hundred Indians, whom it referred to as ‘native dressers’ or ‘native doctors.’^17^ Official documents describe them as ‘Natives, who have acquired some knowledge of preparing Medicine, Dressing wounds, and waiting on sick.’^18^ Mukharji states that they were generally recognised as Eurasians or Brahmins.^19^ In other British colonies, such as Canada, the position of dresser also existed but referred to senior medical students who performed similar functions. Native dressers in India were hospital orderlies, not medical students. Their status, conferred by the colonial category of ‘native,’ seems to have been specific to the medical relations that emerged under the BEIC. Those who held the title of ‘native dresser or doctor’ were comparable to what Cristiana Bastos, in her study of the medical school in Goa, has called a ‘subaltern elite,’ that is, ‘a local elite, either descended from European colonisers or members of Hindu [or Muslim] upper strata. They were also a cosmopolitan elite when living in other colonies. And yet they were a subordinate group’ that could ‘not enjoy the career privileges of those trained in [Europe]’.^20^ This echoes the ‘paradoxical position’ of colonial-trained doctors analysed by Hans Pols and Hohee Cho in their contributions to this issue.^21^

The hospitals and dispensaries where the native dressers worked allowed for the observation of patients and the autopsy of unclaimed bodies, mainly of Europeans, by doctors or surgeons.^22^ Indians of the lower caste of Dom were employed as assistants for post-mortem examinations, which, according to David Arnold, indicates the ‘deep repugnance’ that dissection aroused in the Indian population.^23^ High mortality rates, loose rules about the disposal of the dead, the usual absence of relatives to claim the bodies and the desire of European practitioners to understand the diseases that posed a costly risk to the troops stationed in India facilitated post-mortem examinations in these colonial institutions. Many of the British practitioners working there had been trained in Edinburgh or London, where experimental and empirical approaches to medicine were widely valued. As in Canada, Scottish practitioners formed a large medical diaspora in India and contributed to introducing post-mortem examinations in medical education.^24^

It is important to note that these approaches to understanding pathological lesions through the opening of a significant number of bodies, although increasingly well established in Paris and a few other cities from the beginning of the nineteenth century, were then only practised in some British medical schools, notably Edinburgh, but not so much in older faculties, such as Oxford or Cambridge, where medical education was still largely based on the study of canonical texts. It is, therefore, difficult to speak of British standards of medical education for this period, but it seems that the looser regulation of medical practitioners in the colonies, and the fact that many of these practitioners had been trained in schools that taught pathological anatomy, encouraged more experimental approaches to medical education in the colonies.^25^

Why were they interested in human remains? For some practitioners, such as James Annesley, the study of sick or dead bodies in India stemmed from an interest in the effects of ‘hot climates’ on unacclimatised bodies. The idea that Indian and European bodies were somehow different underpinned this interest.^26^ This emphasis on the link between climate and health, as Partho Datta argues, is ‘built on the idea that […] Europe had their roots in temperate climate and geography. […] In the eighteenth and nineteenth centuries, a period of ascendant nation-states in Europe, this was of course a great boost to national vanity.’^27^ Learning to adapt physically to new environments led British practitioners to publish medical topographies of India.^28^ Their concern about the climate in India may have been exacerbated by the terrible cholera epidemic that spread from Jessore between 1817 and 1821.^29^

Medical training involving dissection and therapeutic experimentation was therefore available in British hospitals and dispensaries in India before the Calcutta Medical Board took the initiative to set up a proper medical school in 1822, which opened the following year. This school, called the Native Medical Institution (NMI), was designed to train young men from the Indian elite in aspects of British medicine in order to provide medical staff for the BEIC at a low cost.^30^ Funding for such educational institutions in India was provided for in the East India Act (1813).^31^ According to Samarendra Nath Sen, the NMI was established when the British needed more doctors for the sepoy regiments, at a time when the BEIC’s general hospitals had been abolished and Indian medical assistants were no longer being trained.^32^

In Bengal, this was a period of ‘medical eclecticism’ marked by a degree of interaction between Indian and British practitioners. To use Projit Mukharji’s phrase, the NMI coexisted in Calcutta’s mixed ‘medical market’ with medical apprenticeships and pre-colonial health traditions that included Indians or Europeans in various initiatives.^33^ While the medical school remained small, it was to represent this eclecticism by incorporating elements of British, Ayurvedic and Unani health care practices.

As Seema Alavi has shown, the Calcutta Madrasa offered lessons in Unani and Ayurveda as early as the 1780s, but separately, which meant that only Muslims studied Unani in Arabic and Hindus studied Ayurveda. This was in keeping with ancient aristocratic traditions of scholarly study. In contrast to the Calcutta Madrasa, however, the NMI aimed to create a new class of civil servants who would navigate between the British and Indian medical worlds and look after the health of every individual. The model proposed at the NMI, according to Alavi, broke with the aristocratic component of the Persian medical tradition inherited from the Mughal Empire by introducing anatomical references that were supposed to be universal, levelling out differences between individuals and challenging social hierarchies. In addition, Urdu, a popular language, was chosen as the common language of instruction for the students enrolled at the NMI, most of whom did not come from traditional medical families. The novelty of the NMI was the desire to provide a medical education that was open to both Unani and Ayurveda, to Hindus and Muslims and that was not based solely on texts and family traditions. According to Alavi, the NMI made the teaching of medicine in the Calcutta Madrasa increasingly obsolete. It also created competition between established medical families and the new category of doctors it was training.^34^

At that time, Indian and British practitioners still found common ground in the humoral understanding of disease.^35^ However, medicine in some medical schools in Britain was undergoing a surgically inspired shift towards pathological anatomy – the study of lesions on diseased and dead bodies. Although it was intended to incorporate certain elements of Indian traditions, the NMI in Calcutta emphasised these new approaches. From being considered a rather dishonourable practice associated with knife trades, such as butchers and barbers in Britain, human dissection had been on the rise as a criterion for defining competent medical practitioners. In the background was the influence of the medical environment in Paris, which attracted large numbers of British students. A competent surgeon or doctor would soon become one who ideally dissected a human cadaver himself in order to understand its constitution and, by the first half of the nineteenth century, one who trained his eye to detect signs of disease outside as well as inside the body.^36^

A parallel shift was seen in the increasing emphasis on chemistry in British medical education. More and more learned men in the natural sciences were isolating plant alkaloids, such as morphine, strychnine, caffeine and quinine, in the laboratory in order to standardise their use, particularly in medicines. In 1822, the French doctor François Magendie published his influential *Formulaire pour la préparation et l’emploi de plusieurs nouveaux médicamens*, in which he summarised how some alkaloids could be isolated and put to pharmaceutical use. This influential treatise was quickly translated into several languages, including English.^37^

The NMI in Calcutta, along with metropolitan institutions such as the University of Edinburgh, served this redefinition of medicine. The NMI acquired anatomical plates, models and treatises, as did all the medical training institutions set up by the British administration during the nineteenth century, and included chemistry in its curriculum.^38^ The NMI students were taken to the general hospital and the BEIC dispensaries to attend dissections by British practitioners and gain clinical experience, as well as perform animal dissections and study illustrations, models and anatomical specimens.^39^ From the 1830s onwards, a number of Indian students were employed to prepare and assist in the dissection of bodies. To the extent that these elements were deemed useful and compatible with imperial priorities, the NMI was also designed to incorporate elements of Indian medical traditions into its teaching.

The NMI in Calcutta was headed by the surgeon Peter Breton from October 1823. A translator and Orientalist, Breton was known for writing treatises for students that he co-authored with Indian scholars and translators. One such publication was a glossary of medical terms translated into English, Arabic, Persian, Hindi and Sanskrit.^40^ Breton introduced his book by praising Indian remedies, saying that some are ‘superior to those of European Physicians.’ On the other hand, he thought that such remedies must be used in accordance with what he called the ‘science of medicine and anatomy’ in order to make them ‘uniformly beneficial to mankind.’^41^

Breton’s successor as director of the NMI, John Tytler, also championed the teaching of anatomy and later established a thirty-bed hospital for the clinical training of students in collaboration with the Bengali intellectual and banker Ram Comul Sen.^42^ For some British practitioners, advocating anatomical dissection was tantamount to reviving Indian medical traditions, as they lamented that the experience of dissection described in ancient Hindu treatises had in their view been forgotten.^43^

The extent to which the NMI offered a genuine blend of Indian and British medical approaches has been debated. If Breton’s account is to be trusted, some British officers thought that Indian remedies were superior to those they could produce themselves. However, they wanted to make their use conditional on anatomical training and experimental chemistry. The potential they saw in some aspects of Indian medicine apparently remained subordinate to their own expectations and agendas.

According to its admission records, the NMI in Calcutta recruited 26 students in its first two years of operation, many of whom came from distant areas. The school appears to have achieved its goal of enrolling both Muslim and Hindu young men, as the title Shaikh precedes the names of five students, presumably indicating that they were Muslims, while Singh is the surname of four other students, suggesting Sikh or Hindu background.^44^ Graduates were expected to serve in the BEIC’s sepoy regiments. Coincidentally, the first Anglo-Burmese War broke within two years of the school’s opening. Criticism in Britain about the cost of this war must have reinforced the imperial administration’s belief that it was better to train Indian medical officers cheaply and employ them at low wages than to rely on British-trained practitioners to care for the troops stationed in Bengal.^45^

The war may also have contributed to the opening of a second NMI in January 1826, this time in Bombay.^46^ The Education Department of the Bombay Presidency, headed by Mountstuart Elphinstone, proposed to its Medical Council the idea of promoting British medical education among the ‘natives’ and encouraging the translation of medical treatises into ‘native languages.’^47^ In response to this idea, the General Medical Council pointed to the teaching of anatomy as the main reason for its reservations. It stated that because of ‘the impossibility of conveying to them [Indian students] an acquaintance with anatomy and consequently with clear physiological and pathological notions their progress in the higher branches of the profession must be slow and limited.’^48^ British practitioners and administrators assumed that Hindus abhorred dissection, a claim that Peter Breton had nuanced, perhaps seeking to protect his own school from criticism.^49^

The Bombay Medical Council suggested that a short bedside apprenticeship in a hospital, focusing on basic remedies and techniques such as bloodletting, should be combined with medical school training in ‘materia medica […] anatomy, physiology, chemistry and perhaps botany,’ and lessons in physics and surgery.^50^ It predicted that upper caste graduates would set up private practices and create medical dynasties.^51^ Instead, the governor decided that the Bombay school should be modelled on the NMI in Calcutta. Its constitution, therefore, stipulated that it would admit at least twenty Muslim or Hindu upper caste students aged between fifteen and twenty-two, speaking either Hindustani, Marathi or Gujarati. They would be enrolled as soldiers and serve for fifteen years in the civilian or military branches of the Indian Medical Service after completing their studies.^52^

The superintendent (and only professor) of the Bombay school was the twenty-five-year-old Scottish surgeon John McLennan. Together with two Indian munshis and a peon, he set about translating treatises for the students, particularly on human anatomy. McLennan imported anatomical specimens and models from Britain and planned to organise clinical teaching at the local hospital. He also procured ‘a small set of chemical apparatuses […] to show some of the common pharmaceutical processes.’^53^

However, both NMIs were soon called into question by the Court of Directors of the BEIC due to recruitment problems and the perception that they were not living up to initial expectations and were not worth the cost. As early as 1826, the Calcutta NMI faced the prospect of abolition. While defending the institution, its superintendent gave a mixed report on its operations.^54^ On the one hand, he praised the students who had helped during a cholera outbreak in the Arakan region and in Calcutta, ‘distributing medicine […] and affording to the wretched and numerous victims of that disease every assistance in the power of European Art.’^55^ He added that ‘native doctors’ were more accessible to the Indian population than their European counterparts. In a letter to his colleague John Gilchrist, the superintendent made it clear that the ‘European art’ he was referring to was based on anatomical knowledge:

You, who have been in India, are well aware of the acquirements of the Native medical practitioners. Their knowledge of anatomy borders on nonentity, and their skill in physic is not far above their anatomical knowledge. What a blessing then it will be to the Natives generally, to have amongst them their own countrymen, educated on system to the medical profession, and capable of alleviating human affliction, which at present consigns to a premature grave myriads of deceased inhabitants of our Eastern empire.^56^

Members of the Bengali elite, such as the head of the Calcutta conservative Hindu society, Radhakanta Deb, and Hindu reformer Ram Mohan Roy, were also quoted in official documents as praising the school and opposing its closure.^57^

On the other hand, the superintendent cited recruitment difficulties. He suggested lowering the age of admission to fourteen and offering students an annual stipend of ten rupees, rising to twelve rupees after two years. This, he thought, would discourage prospective students from opting to settle down and start a family rather than embark on a long medical course. He also recommended that the institution create permanent posts for two Hindu and two Muslim students. The government’s military department accepted these recommendations the following month.^58^

In Bombay, too, the medical council quickly lost confidence in the local NMI. Of the seventy students who had been admitted, thirty-five were expelled because, according to one report, they were deemed unfit or insufficiently committed to learning medicine. Another nine students left voluntarily before graduation.^59^ The board cited inadequate translation of medical treatises and the superintendent’s failure to provide students with appropriate hospital and anatomy instruction. To save money, in 1829, the governor asked that the services of a munshi be dispensed with and that the number of students be reduced to ten. In addition, to discourage students from leaving the school, he ordered that the scholarship paid to students be increased.^60^

Despite this, the Bombay Medical Council concluded that the school did not provide sufficient benefits when compared to the training of ‘native doctors’ in the colonial hospitals. In addition, one member of the Board felt that the Indian population showed little interest in consulting BEIC-trained doctors, limiting the ability of graduates to earn a living through private practice.^61^ As a result, the Bombay NMI was closed on 21 December 1832 after six years of operation. Following the report of a committee appointed by the Governor-General of Bengal, the NMI in Calcutta was also closed in 1835, having trained a total of 166 doctors since its inception.^62^

On the whole, it is clear that the establishment of the NMIs in Calcutta and Bombay followed a top-down approach initiated by the imperial administration, and, more specifically, by the Medical Boards of the Presidencies. However, this approach was not replicated in at least one case in India during this period – a case that has been overlooked by historiography. In 1834, Naṣīruddīn Ḥaidar, the King of Awadh (the neighbouring state of Bengal, called Oudh in British sources), offered the British government in Calcutta a loan of 350,000 rupees at four percent interest to establish a British-run hospital and medical school in Lucknow. In his request, King Ḥaidar explained that the school would compete with the one in Calcutta and meet the growing demand for doctors in his region, where he had established a hospital less than two years earlier. On 13 March 1834, Secretary Charles Trevelyan replied positively on behalf of the Calcutta government.^63^ The agreement stipulated that the King of Awadh’s British surgeon would supervise the hospital and give medical lessons to the students. A recognised practitioner would also be appointed to train hakims in Unani at the hospital. This is an example of a British medical school project that would have been funded by a local Indian authority. It is difficult to know whether this plan came to fruition or not. It may not have survived the death of King Ḥaidar three years later, who was succeeded by another dynasty.^64^

It is quite possible that the King of Awadh’s proposal was intended to show that the region met the British criteria for modernity. By proposing this school, King Ḥaidar may have hoped to avoid having reforms imposed on his region by the British imperial administration and to retain some autonomy of government. Although a vassal, the Kingdom of Awadh was not under the direct control of the BEIC. The royal family was still recognised by the British. However, the threat of annexation may have prompted the king to seek the BEIC’s favour. His offer to fund a medical school came only a few years after the governor-general issued an initial annexation order for the region, which had not been carried out. However, these efforts were ultimately in vain. The Awadh region was annexed in 1856 by order of the Governor of the BEIC.^65^

## British medical education in Canada (1763–1837)

Examining a parallel colonial history on the other side of the globe strengthens the claim that a global negotiation of standards in medical education was beginning to emerge in the British Empire in the early nineteenth century. It also shows that there was no single route to implementing similar core disciplines in medical education across continents. Although not the result of a single policy from the imperial capital, nor of similar conditions or intentions in each territory, some core standards of medical education were, however, negotiated in Asia and the Americas in the context of the British Empire. The relative standardisation of British medical education depended on a changing set of often unrelated factors in different parts of the empire.

In India, it made economic sense for the imperial government to train doctors locally and employ them at low cost. The risks and expense of travelling to and from Britain were such that recourse to British practitioners was not very effective. Moreover, India was important to the empire in commercial, military and geopolitical terms. Canada, on the other hand, does not seem to have been as important to the imperial authorities, who seemed to see it more as what remained of the British colonial territory in North America in the aftermath of the American Revolution. British doctors and surgeons in Canada enjoyed greater autonomy, more opportunities to acquire property and less competition in the provision of health care than their counterparts stationed in India. They also benefited from shorter, less strenuous and less risky journeys to connect with the imperial metropole. It follows that, unlike its development in India, British medical education in Canada was not the result of initiatives by the imperial authorities. Rather, it was defined by transatlantic networks that linked Montreal and Quebec City primarily with London and Edinburgh but also with Paris, Boston and New York. The content of British medical education in Canada bore striking similarities to that which was being institutionalised in India at the same time. This shows how the institutionalisation of medical education in medical schools and the establishment of a more explicit and uniform curriculum are historically intertwined.

Towards the end of the eighteenth century, military surgeons stationed in North America during the imperial wars, particularly the American Revolutionary War, settled in the Canadian colony and earned a living, in part, by training apprentices. In the following decades, practitioners trained in London or Edinburgh, some of them influenced by Paris medicine, took the initiative to establish medical schools in Canada in order to regulate medical practice more strictly, to meet the needs created by urban poverty and to limit the number of Canadian students enrolled in medical schools abroad. Rainer Baehre has shown that some of the founders of the first medical schools in Canada had a political interest in keeping medical students away from the United States, whose republicanism was widely distrusted in Canada after the War of 1812.

As in India, the institutionalised medical curriculum in Canada emphasised human dissection, clinical training in hospitals and organic chemistry. However, in contrast to its centrality in British medical research in India, the issue of physical acclimatisation does not seem to have been considered very important in Canada. Dissecting the human body was valued in medical training but does not appear to have been used to understand how the climate affects the body, at least not as it was in India. In many ways, the North American environment appeared to British settlers as an extension of the metropole. Medical discourses on the relationship between climate, physical constitution, race, environment and disease seem to have been even less prominent in Canada than in the United States, perhaps because Canada was still part of the British Empire and did not really have the same overseas ambitions as its southern neighbour, as discussed by Christopher D. E. Willoughby in this issue. It is possible that the territorial expansion of Canada towards the Pacific Coast led to the use of such discourses in medical education, but this would be a matter for further research.

The economic, political and military stakes of the Canadian colony were much lower from the British imperial point of view than those of India, which meant that the imperial authorities took far less initiative and devoted fewer resources to medical education in Canada than in India. As a result, medical education in Canada was largely based on private apprenticeships until well into the nineteenth century. The first medical schools were the result of bottom-up rather than top-down initiatives. These schools were largely locally funded and subject to a laissez-faire attitude on the part of the imperial administration. Urban, wealthy, English-speaking, Protestant local elites loyal to the British Crown became the main driving force behind the first medical schools established in Canada. A relatively new British colony, Canada was ceded to the British Crown in 1763 after being a French colony (*Nouvelle-France*) for two centuries. Before 1788, according to Rénald Lessard, about ten licensed physicians and 500 surgeons, most of them French, had settled in the colony.^66^ Before the British took over the colony, hospitals such as the Hôtel-Dieu in Montreal and Quebec City were founded by French Catholic communities. They were far from the research and teaching institutions they would become in the nineteenth century.

Under the new British regime, the Canadian colony was ruled by a governor-general without an administrative structure on the scale of the BEIC. The indigenous peoples living in the territory were soon outnumbered by the European settlers. Although there had been some exchange of knowledge about remedies and medicinal plants between the French settlers, particularly the Jesuit and Récollet missionaries, and the indigenous peoples during the seventeenth and eighteenth centuries, this exchange seems to have diminished after the colony came under British rule.^67^ In any case, no attempt was made in Canada to integrate pre-colonial health practises into British medical education, and there seems to be no indication that imperial officials regarded Canada’s indigenous peoples as a civilisation from which they could usefully learn, as some did for India and its medical traditions.^68^

It is obviously easier to travel to Europe from Canada than from India. Thus, from the second half of the eighteenth century, many Canadian apprentices crossed the Atlantic to study medicine, particularly in Edinburgh, London and Paris, while at the same time, some graduates of European institutions settled in Canada. In its reliance on transatlantic networks, medical education in Canada resembled that in other British North American colonies, including Jamaica and Trinidad, albeit with distinctly different racial dynamics.^69^ It was not until 1845 that a doctor fully trained and licensed in Canada helped to establish a medical school in the colony.^70^

As a result of transatlantic exchanges in medical education, practitioners in Canada became involved in the dissection of human bodies.^71^ But there seems to have been no attempt in this study of human anatomy to investigate the relationship between climate and disease, unlike the autopsies carried out in what the British called the East and West Indies, roughly the Caribbean and India. In other words, climate does not seem to have been considered a major medical threat in colonial Canada, unlike areas classified as ‘hot’ or ‘tropical,’ despite having harsher winters than Britain.^72^ In his *Observations on Emigration to British North America* (1829), the Scottish politician and statistician John MacGregor went so far as to write that the ‘*climate of British America is too salubrious for doctors to make fortune*’ (italics in the original source for emphasis).^73^ To European practitioners, the Canadian flora, fauna and landscape formed what Christopher Parsons aptly called a ‘not-so-new world’ and was therefore considered more hospitable.^74^

The first – not very successful – attempt at government regulation of medical practice in Canada came on the advice of two military surgeons, James Fisher and Charles Blake. The content of these surgeons’ own medical training in Britain prior to their arrival in Canada is unclear. What is known is that both had been stationed in British North America to assist the imperial forces during the American Revolutionary War and had subsequently settled in the colony. Fisher and Blake echoed calls from Britain for a clearer distinction between legitimate practitioners and ‘fringe medicine.’^75^ In 1784 and 1786, respectively, they wrote to the committees appointed by the governor-general to find ways of promoting the growth and health of the colony’s population. Their letters identified surgery, physics, materia medica and obstetrics as crucial disciplines for the colony and deplored the incompetence of ‘those pests to society,’ the ‘empirics,’ a term often used to refer to healers and sellers of remedies suspected of charlatanism. Fisher warned that these ‘unprincipled and illiterate pretenders to science’ were endangering the health of the population and suggested that the imperial administration should set up some sort of professional body to check the qualifications of those wishing to practise medicine or surgery in the colony.^76^ More specifically than Fisher, Blake cited instances of malpractice that he claimed caused the deaths of patients during the St Paul’s Bay epidemic (1782-c. 1791).^77^ According to historian Rénald Lessard, Fisher and Blake were probably targeting German surgeons who had been sent to the colony as mercenaries during the American Revolutionary War and remained active in Canada afterwards.^78^

In 1788, following Fisher and Blake’s recommendations, the Governor-General of Canada ordered the establishment of medical licensing boards in Montreal and Quebec City.^79^ These boards remained weak until the mid-nineteenth century. Upper Canada (roughly the southern part of present-day Ontario) did not have an effective medical board until 1827, as no fewer than six medical acts were passed between 1795 and 1865 to regulate the practice of medicine, while in Lower Canada (a part of what is now Quebec), it was in 1847 that a College of Physicians and Surgeons was created.^80^ For thirty years after the governor-general’s order of 1788 on Boards of Examiners, colonial medical education in Canada was conducted exclusively through private apprenticeships.

The institutionalisation of medical education began in 1818, when a dispensary opened in the port city of Quebec, mainly to treat immigrants. In the wake of the Napoleonic Wars and the War of 1812, passenger ships arrived daily from the Atlantic. This institution was modelled on the Scottish dispensaries. It provided training in the principles and practice of medicine, anatomy, surgery, physiology, midwifery and anatomy. Its founders had studied in Edinburgh, in London hospitals or at Harvard Medical School, all of which offered similar medical training. Difficulties of an unspecified nature led to the closure of the dispensary just over a year later.^81^

Soon after, physicians trained in New York, London, Edinburgh or Paris opened an emigrant hospital in Quebec City, which from 1823 provided undergraduate training in the theory and practice of medicine, surgery and midwifery, apparently until the early 1840s.^82^ Meanwhile, in Upper Canada, London-trained John Rolph and New York-trained Charles Duncombe established the Talbot Dispensary at St Thomas (near Lake Erie) under the patronage of Irish-born Colonel Thomas Talbot.^83^ Opened around 1824, their school offered training in the theory and practice of medicine, surgery, physiology and anatomy to a number of apprentices, apparently too small for the school to remain active for more than two years. None of these institutions awarded medical degrees.

Alongside these institutional initiatives, the standards of medical education that were gaining ground in the empire took root in Canada through networks of amateur scientists and public lectures.^84^ Amateur scientific societies in Montreal sometimes gave lectures on chemistry with practical implications for medicine.^85^ Individuals trained in science and medicine also offered public lectures that were not part of an institutionalised curriculum. For example, William Willcocks Sleigh (1796–1863), an Irishman educated at Trinity College, Dublin, settled in Montreal for four years (1819–1823), during which he gave lectures on physics, materia medica and the principles of chemistry, as well as demonstrations of anatomy, physiology, surgery and clinical practice. Alexander Ramsay, a physician and former lecturer in anatomy, physiology and natural science in Edinburgh, and Andrew Smyth, a former military surgeon, also gave public lectures in Montreal at the turn of the 1820s, particularly on anatomy and surgery.^86^

Canada’s first degree-granting medical school was established in the late 1820s as an offshoot of the Montreal General Hospital. Originally called the Montreal Medical Institution (MMI), it educated students through lectures and on the wards. The MMI offered courses in anatomy, physiology, surgery, chemistry, pharmacy, botany, materia medica, dietetics, practical medicine, midwifery and women’s and children’s diseases, in line with the training received by its founders in Edinburgh, London and Paris.^87^ As such, it was designed to provide students with a comprehensive and up-to-date medical education without having to leave the city. Its establishment was made possible by a close-knit social and philanthropic network of British business families known as the ‘Château Clique.’^88^ In other words, unlike the NMIs in Calcutta and Bombay, this school in Montreal was not an initiative of the official colonial authorities. Nor was it first established as an independent school before being attached to a hospital. Rather, it emerged as a subdivision of an existing hospital, privately initiated by local elites.

The Montreal General Hospital, where the MMI operated, was founded by women from the city’s English-speaking and Protestant business community who came together to form the Female Benevolent Society. They first opened a soup kitchen, a children’s education room and a four-bed hospital around 1818 to care for the growing number of immigrants who arrived in Montreal weakened by hard travel and lacking resources and shelter. Demand soon outstripped capacity, and subscriptions transformed the initiative into a twenty-four-bed hospital run by local businessmen. By the early 1820s, the hospital’s connection with the city’s Masonic Lodges and Anglican or Presbyterian places of worship (the sociability of the male Protestant elite) was clear.^89^ This was the environment in which the MMI took shape.

Despite its Protestant administration, the Montreal General Hospital was, in principle, open to patients of all faiths and suffering from all diseases, especially destitute immigrants. By 1822, the hospital had moved to larger premises, and it was there that the MMI began its work as an independent department of the hospital – the same year, incidentally, that the NMI was established in Calcutta.^90^ The Governor-General of British North America (later Commander-in-Chief of India), Lord Dalhousie, quickly appointed the five founders of the MMI to the Board of Medical Examiners in Montreal. As a result, these doctors gained the upper hand in medical licensing in the city, and their school became the fastest way to enter the profession in Canada until the 1830s, with the other ways being to graduate from a medical school abroad or to take the medical board examination after completing a private apprenticeship.^91^ The MMI could not award degrees because it was not yet affiliated with an accredited university. Graduates had to pass the city’s medical board examination.

The MMI only became Canada’s first degree-granting medical school when it was incorporated into the newly formed McGill College. In 1813, Scottish-born businessman James McGill died in Montreal and bequeathed the estate of his summer home to the Royal Institution for the Advancement of Learning (RIAL) to establish a college, provided it was operational within ten years. In doing so, James McGill was fulfilling the mandate of the RIAL, which had been created twelve years earlier to ensure the establishment of educational institutions in Lower Canada.^92^ Although McGill College was granted a Royal Charter in 1821, thus securing James McGill’s estate, it still existed only as a legal entity and had not begun to teach. It needed to attract professors before it could be inaugurated as a proper university.^93^ The MMI, for its part, was denied a charter and had to affiliate with an existing university or college to gain the right to confer degrees, hence its merger with McGill College in 1829.^94^ Like other medical training institutions in the British Empire, the resulting McGill Faculty of Medicine emphasised bedside teaching, human dissection and organic chemistry, following the example of the Edinburgh Faculty of Medicine but also, as the historian Richard Vaudry has shown, that of Paris.^95^

While it would be anachronistic to speak of medical specialties so early in history, we do see the emergence of practitioners whose specific skills were reflected in medical curricula and regulated by official medical authorities. This was the case, for example, with midwifery, particularly in a context such as that of Canada, where the colonial administration was interested in population development and, therefore, in medicine that could assist in childbirth. In 1833, for example, ‘Mrs Baptiste Barette’ was registered as a midwife (‘*sage-femme*’) after passing the examination of the Montreal Medical Board.^96^

McGill’s Faculty of Medicine remained rooted in the circles of Canada’s Protestant (mainly Anglican) elite. Thirteen clergymen were present at the official opening ceremony. Several prayers and scriptures were read.^97^ The principal of McGill College was George Josaphat Mountain, the Norfolk-born Anglican archdeacon of Quebec City at the time of MMI’s integration. Mountain had volunteered to fight on the British side against the United States in the War of 1812 and had also helped lay the foundations for a school system in New Brunswick through the Society for Promoting Christian Knowledge around 1815.^98^

Although not present at the inauguration, the Archdeacon of York (in present-day Ontario) and future Bishop of Toronto, John Strachan, was an honorary professor of history and civil law at McGill College when it merged with the MMI. He had been instrumental in the founding of the college. A close friend of James McGill and one of the trustees of his estate, Strachan also married McGill’s widowed sister-in-law, Ann Wood McGill.^99^ He had founded and chaired the Loyal and Patriotic Society of Upper Canada to support British troops in the War of 1812 against the United States. Through this society, he also helped to finance and build what became the Toronto General Hospital, which opened in 1829.^100^ A prominent member of Upper Canada’s Executive Council for some fifteen years, Strachan served on the legislative council for nearly a decade. He was, thus, one of the key leaders of an influential local elite in Upper Canada known as the ‘Family Compact,’ largely made up of descendants of Loyalists who had fled the United States during the Revolution.^101^

As Rainer Baehre argues, one of Strachan’s main reasons for supporting McGill College was his concern about the spread of republicanism in Canada. In other words, Strachan feared that without a local option, young Canadians seeking an education would go to the United States, where republican ideas could make them a threat to the British Crown on their return. For similar reasons, Strachan led a project to establish a King’s College in Toronto, which had been in the works since the United States gained its independence.^102^ In 1827, he obtained a Royal Charter for Toronto’s King’s College, which stipulated that it should be presided over by a member of the United Church of England and Ireland. Strachan was the first to hold this position. Initially, King’s College Toronto was so closely associated with Loyalist circles and the Anglican clergy that opposition delayed its construction until 1842, after the charter was amended to defuse Anglican influence. It thus became the first medical school to grant degrees in the region of Upper Canada.^103^

Similar to McGill’s Faculty of Medicine and other medical schools in the empire, including those in India, the medical curriculum at King’s College Toronto was based on theoretical and practical anatomy taught by Henry Sullivan, a Fellow of the Royal College of Surgeons in London, and William Charles Gwynne, a Bachelor of Medicine from Trinity College, Dublin. In line with educational standards emerging across the British Empire, students were required to take courses in chemistry, the theory and practice of medicine, the principles and practice of surgery, midwifery, women’s and children’s diseases, materia medica and pharmacy.^104^

It is unclear which texts were used for medical education in Montreal and Toronto at the time. A systematic study of the production, circulation and use of medical texts in the British Empire remains to be undertaken. Ships’ cargo inventories, as well as posthumous inventories of individuals’ possessions can be interesting sources in this regard. Inventories of this kind kept in Pondicherry, for example, reveal some of the medical books that were available in this French colony in India at the end of the eighteenth century.^105^

## Conclusion

In the early 1830s, a student at a British medical school in Montreal and one studying at a colonial medical school in Calcutta or Bombay would receive similar training in many respects. This kind of negotiated standardisation was seen in Europe and the United States, and it went much further, across empires.^106^ Under the jurisdiction of the British Empire, the colonies of Canada and India began a process of institutionalisation of medical education around core disciplines that remained relatively modest but would gain momentum during the nineteenth century. The NMIs in Calcutta and Bombay were established to train what imperial officials called ‘subaltern doctors.’ In Canada, the War of 1812 proved decisive in the creation of medical teaching institutions that would allow Canadians to complete their education in their own territory. These Canadian schools were also seen by some of their founders, such as John Strachan, as a means of preventing Canadians from studying in the United States and being exposed to republican ideology, thus protecting what remained of the British presence in North America after the American Revolution. Understandably, direct communication between Canadian and Indian medical circles was not common at the time. However, the example of George Ramsay, Earl of Dalhousie, who served successively as Governor-in-Chief of British North America (1820-1828) and Commander-in-Chief of the British army in India (1828-1832), illustrates how political figures could move from one colony to another in the empire. Doctors were also interested in other parts of the empire, as evidenced by Joseph Workman’s doctoral dissertation, submitted to the Faculty of Medicine at McGill College in Montreal in 1835, which traced the history of the ‘Asiatic cholera’ epidemic from Jessore in India in 1817 to Canada in 1832.^107^

This article has sought to highlight the role of the British Empire in the globalisation of standards in medical education from the late eighteenth century. This process was neither uniform nor linear. In fact, the intentions that led to the establishment of the first British medical schools in India and Canada were set aside as early as the Victorian era (1837–1901). The inclusion of elements of Ayurveda and Unani in British medical education was abandoned in India as soon as the NMIs in Calcutta (1835) and Bombay (1845) were replaced by institutions based on a stricter British model and teaching only in English – as was a new medical school established in Madras (1835).^108^ Former employees who taught Ayurveda or Unani in the NMIs, such as Hakim Abdul Majeed, were denied employment in the new colleges because they did not speak English.^109^ The colonial government of the day in India generally imposed the English language in its administration.^110^

The recruitment capacity of the new Calcutta Medical College proved to be greater than that of the NMI, with 456 ‘native doctors’ trained in Calcutta alone between 1835 and 1858.^111^ The colonial administration boasted loudly that in 1836, an Indian demonstrator, Pandit Madhusudan Gupta, and four Indian students, Umacharan Set, Rajkrishna De, Dwarakanath Gupta and Nabin Chandra Mitra, had performed the first human dissection in this school.^112^ The colonial authorities celebrated the event as a symbol of the definitive introduction of British medicine to India.^113^ The Calcutta Medical College was officially supported by the Royal College of Surgeons of England from 1845, and as Mark Harrison writes, ‘was supplied with 3,500 Indian bodies for dissection between 1837 and 1847,’ and ‘established a pathological museum that drew thousands of specimens from around the country to teach students the morbid signs of diseases.’^114^ Some of the former students and staff of the NMIs went on to teach medicine in rural areas of India.^115^ The interaction between Indian and British medicine continued to some extent in the BEIC dispensaries.^116^

Meanwhile, the cholera epidemic that had ravaged India since 1817 arrived in Canada in 1832 and reshaped medicine with investments in quarantine stations and new health facilities.^117^ The failed attempt at a republican revolution in Canada in 1837–1838 also marked a turning point, after which the Canadian medical profession developed greater autonomy from the British imperial administration. Many doctors fought on both sides of this armed conflict.^118^ Governor-General John George Lambton Earl of Durham’s report on the uprising led to governmental changes that resulted in the loss of influence of the Anglican elites who had spearheaded the establishment of the first medical schools in Upper and Lower Canada.^119^ The defeat of the revolt also created a political vacuum that was quickly filled by the Catholic Church. From the 1840s, francophone medical schools founded or sponsored by Catholic clergy – the *École de médecine et de chirurgie de Montréal* and the *École de médecine de Québec*, both incorporated in 1845 – permanently changed the dynamics of the medical profession in Canada.^120^ The College of Physicians and Surgeons of Lower Canada emerged as a new professional regulatory body in 1847, eleven years before its British counterpart.

Since then, India and Canada have followed very different paths in their relationship with the British Empire and its aftermath. India underwent a process of independence that culminated in the Constitution of the Republic of India in 1950. Canada, on the other hand, constituted itself as a confederation in 1867. Despite latent republican tendencies and two referendums on Quebec’s independence (in 1980 and 1995), Canada remains a parliamentary monarchy headed by the British monarch, as do Australia and New Zealand. Both India and Canada are now members of the Commonwealth. In recent decades, doctors trained in India have moved in large numbers to take up positions in the United Kingdom’s National Health Service, while there has been no comparable phenomenon among Canadian doctors.^121^

Nevertheless, the early colonial medical schools of the British Empire discussed in this article continue to shape our medical world. The Calcutta Medical College and McGill University’s Faculty of Medicine, for example, are not only still active but are among the most prestigious educational institutions in their respective countries. Their centuries-old cosmopolitanism continues to influence student enrolment in a medical world that is more international than ever, even as the imperial past of these medical schools comes under renewed scrutiny in current debates about colonial legacies in the contemporary world.

## Footnotes

1 I am grateful to the participants in the Oxford workshop from which this article is derived, especially Mark Harrison for his comments as discussant and Erica Charters, who supervised my research. I would also like to thank the editors and anonymous referees of *Medical History*, as well as Seema Alavi who have contributed greatly to the improvement of this text. The research that made this article possible was supported by the Social Sciences and Humanities Research Council of Canada, the *Fonds québécois de recherche – Société et culture*, the *Institut francilien recherche, innovation, société*, and the Faculty of History as well as Wolfson College at the University of Oxford. I am, of course, solely responsible for any shortcomings herein.

2 ‘Professors of Racial Medicine: Imperialism and Race in Nineteenth-Century United States Medical Schools’.

3 As analysed for anatomy in Emmanuelle Godeau , L’esprit de corps. Sexe et mort dans la formation des internes en médecine (Paris: Maison des Sciences de l’Homme, 2007). See also: John Harley Warner and James M. Edmonson , Dissection. Photographs of a Rite of Passage in American Medicine, 1880-1930 (New York: Blast Books, 2009).

4 Rare exceptions include: Michael Zeheter , Epidemics, Empire, and Environments: Cholera in Madras and Quebec City, 1818-1919 (Pittsburgh: University of Pittsburgh Press, 2015); Paula Hastings , ‘Fellow British Subjects or Colonial “Others”? Race, Empire, and Ambivalence in Canadian Representations of India in the Early Twentieth Century’, American Review of Canadian Studies, 38, 1 (2008), 3–26 (the latter does not deal with medicine). Mark Harrison cites the differences between India and Canada as an example that challenges George Basalla’s diffusionist model of science in the colonies: Mark Harrison, ‘Networks of knowledge: Science and medicine in early colonial India, c. 1750-1820’, in Douglas M. Peers and Nandini Gooptu (eds.), India and the British Empire (Oxford: Oxford University Press, 2012), 192 . Although it focuses on students trained in Britain, see also: M. Anne Crowther and Marguerite W. Dupree , Medical Lives in the Age of Surgical Revolution (Cambridge: Cambridge University Press, 2007), Chs. 8 and 9.

5 Respectively, the University of Sydney Faculty of Medicine, and the Faculty of Medical Science of the University of the West Indies (Jamaica).

6 The thesis of a ‘second British Empire’ more oriented towards Asia after the American Revolution has been much debated and is often traced back to: Vincent T. Harlow , The Founding of the Second British Empire, 1763-1793 (London: Longmans, Green, and Co., 1964), 2 vol.

7 Pratik Chakrabarti, *Medicine and Empire: 1600-1960* (London: Palgrave Macmillan, 2013), 58.

8

Pratik Chakrabarti , ‘“Neither of meate nor drinke, but what the Doctor alloweth”: Medicine amidst War and Commerce in Eighteenth-Century Madras’, Bulletin of the History of Medicine, 80, 1 (2006), 10 10.1353/bhm.2006.0009PMC263000416549880.

9 Anna Ruddock and Pratik Chakrabarti, ‘The “Indian predicament”: Medical education and the nation in India, 1880–1956’, in Delia Gavrus and Susan Lamb (eds.), Transforming Medical Education. Historical Cases of Teaching, Learning, and Belonging in Medicine in Honour of Jacalyn Duffin (Montreal and Kingston, McGill-Queen’s University Press, 2022), 164 .

10 Chakrabarti, *op. cit.* (note 8), 3.

11 Mark Harrison and Biswamoy Pati, ‘Social history of health and medicine. Colonial India’; and Partho Datta, ‘Ranald Martin’s medical topography (1837). The emergence of public health in Calcutta,’ in Biswamoy Pati and Mark Harrison (eds.), *The Social History of Health and Medicine in Colonial India* (Abingdon-on-Thames: Routledge, 2009), 5–6 and 17–8.

12

George Thomas Bettany , ‘Harwood, Busick’, in Dictionary of National Biography (London: Smith, Elder & Co., 1885–1900), vol. 25, 101–2.

13

Seema Alavi , ‘Medical Culture in Transition: Mughal Gentleman Physician and the Native Doctor in Early Colonial India’, Modern Asian Studies, 42, 5 (2008), 858–9, 863–4 and 869.

14

Zhaleh Khaleeli , ‘Harmony or Hegemony? The Rise and Fall of the Native Medical Institution, Calcutta: 1822-35’, South Asia Research, 21, 1 (2001), 82 10.1177/02627280010210010418652048.

15

Projit Bihari Mukharji , Nationalizing the Body. The Medical Market, Print, and Daktari Medicine (London and New York: Anthem Press, 2009), 180 .

16

Mark Harrison , ‘Medical experimentation in British India: The case of Dr Helenus Scott’, in Hormoz Ebrahimnejad (ed.), The Development of Modern Medicine in Non-Western Countries (London: Routledge, 2009), 26–38 . See also: Harrison, *op. cit.* (note 4), 200 and 207–8.

17 Mukharji, *op. cit.* (note 15), 2–3 and 59.

18

Conwell, W.E.E. , A Code of Medical Regulations, for the Honorable East India Company’s Establishment of Surgeons, Belonging to the Presidency of Prince of Wales’ Island, Singapore, and Malacca (Singapore: Mission Press, 1823), 2 .

19 Mukharji, *op. cit.* (note 15), 3.

20

Cristiana Bastos , ‘Doctors for the Empire: The Medical School of Goa and its Narratives’, Identities: Global Studies in Culture and Power, 8, 4 (2001), 521 .

21 “The Expansion of Medical Education in the Dutch East Indies and the Formation of the Indonesian Medical Profession”. Hohee Cho’s article is entitled: “Microcosm of the Pacific: Colonial Encounters at the Central Medical School, Fiji”.

22 Harrison and Pati, *op. cit.* (note 11), 5.

23

David Arnold , Colonizing the Body. State Medicine and Epidemic Disease in Nineteenth-Century India (Berkeley: University of California Press, 1993), 4–5 .

24 Mark Harrison, ‘Racial pathologies. Morbid anatomy in British India, 1770-1850’, in Biswamoy Pati and Mark Harrison (eds.), *op. cit.* (note 11), 176; David Arnold, ‘Science, technology and medicine in colonial India’, in *The New Cambridge History of India*, pt. III (Cambridge: Cambridge University Press, 2000), 60; H.E. MacDermot , ‘The Scottish Influence in Canadian Medicine’, The Practitioner, 183 (1959), 84–91 14419300 .

25

Fiona Hutton , The Study of Anatomy in Britain, *1700–1900* (Cambridge: Cambridge University Press, 2013), 111–21; Mark Harrison , Medicine in an Age of Commerce and Empire. Britain and its Tropical Colonies, *1660–1830* (Oxford: Oxford University Press, 2010), part I.

26 Harrison, *op. cit.* (note 25), 173–5; Arnold, *op. cit.* (note 24), 24 and 64.

27 Datta, *op. cit.* (note 11), 20.

28

Mark Harrison , ‘Differences of Degree: Representations of India in British Medical Topography, 1820-c. 1870’, Medical History Supplement, 44, 20 (2000), 55PMC253099411769937. See also: Mark Harrison , Climates and Constitutions: Health, Race, Environment and British Imperialism in India, 1600-1850 (Oxford: Oxford University Press, 2003).

29

Mark Harrison , ‘A Dreadful Scourge: Cholera in Early Nineteenth-Century India,’ Modern Asian Studies, 54, 2 (2020), 503 .

30 Arnold, *op. cit.* (note 24), 54–5.

31

Sunil K. Pandya , ‘Dr John McLennan MD (Aberdeen), FRCP (Lond) (1801–1874) and the Medical School of Bombay that failed’, Journal of Medical Biography, 27, 1 (2017), 47 10.1177/096777201770276228382833.

32

Samarendra Nath Sen , Scientific and Technical Education in India, 1781-1900 (New Delhi: Indian National Science Academy, 1991), 129 .

33 Mukharji, *op. cit.* (note 15), 14–6 and 79–80.

34

Seema Alavi , Islam and Healing. Loss and Recovery of an Indo-Muslim Medical Tradition, 1600-1900 (Basingstoke: Palgrave Macmillan, 2008), 73–6. See also: Khaleeli, *op. cit.* (note 14), 82–5; Sen, *op. cit.* (note 32), 135, 145–6; Jayanta Bhattacharya , ‘The Genesis of Hospital Medicine in India: The Calcutta Medical College (CMC) and the Emergence of a New Medical Epistemology,’ The Indian Economic and Social History Review, 51, 2 (2014), 237 and 241. I thank Seema Alavi for the clarification she gave me on this subject.

35 Harrison, *op. cit.* (note 3), 198.

36 Guenter B. Risse, ‘La synthèse entre l’anatomie et la clinique’, in Mirko Grmek (ed.), *Histoire de la pensée médicale en Occident, tome 2* (Paris, Seuil, 1997), 177–97; Michel Foucault, *Naissance de la clinique* (Paris, Presses Universitaires de France, 1963); Erwin H. Ackerknecht, *Medicine at the Paris Hospital, 1794-1848* (Baltimore, John Hopkins University Press, 1967); Othmar Keel, *L’avènement de la médecine clinique moderne en Europe 1750-1815* (Montreal and Geneva, Presses de l’Université de Montréal and Georg, 2001). See also: Russell C. Maulitz, *Morbid Appearances. The Anatomy of Pathology in the Early Nineteenth Century* (Cambridge, Cambridge University Press, 1987), 18–29; Florent Palluault, ‘Medical Students in England and France, 1815-1858. A Comparative Study’ (unpublished PhD thesis: University of Oxford, 2003).

37

François Magendie , Formulaire pour la préparation et l’emploi de plusieurs nouveaux médicamens (Paris: Méquignon-Marvis, 1821). I wish to thank Catherine Jackson for suggesting this reference.

38 British Library (subsequently, BL), IOR/Z/E/4/42/A468, 18 January 1828; IOR/P/33/6, August 1828, nos. 136–7; IOR/Z/E/4/42/B1216, 24 June 1829; IOR/F/4/1357/54100, 25 October 1831.

39 Khaleeli, *op. cit.* (note 14), 92.

40 ‘Liberality of the Indian Government Towards the Native Medical Institution in Bengal’, *The Oriental Herald*, X, July-September 1826, 17–8.

41

Peter Breton , A Vocabulary of the Names of the Various Parts of the Human Body and of Medical and Technical Terms in English, Arabic, Persian, Hindee and Sanscrit for the Use of the Members of the Medical Department in India (Calcutta: Government Lithographic Press, 1825), 3–4 .

42 Samita Sen and Anirban Das, ‘A history of the Calcutta Medical College and hospital, 1835-1936,’ in Uma Das Gupta (ed.), *Science and Modern India: An Institutional History, c.1784-1947* (Delhi: Pearson Longman, 2011), 483.

43 Arnold, *op. cit.* (note 24), 67–8.

44 BL, IOR/P/31/17, 24 March 1825, 112–3.

45 Alavi, *op. cit.* (note 13), 889–90; ‘Chronology,’ in Andrew Porter and William Roger Louis (eds.), The Oxford History of the British Empire, vol. III: The Nineteenth Century (Oxford: Oxford University Press, 1999), 712–41.

46 BL, IOR/F/4/1357/54154, 3. See also: Sunil K. Pandya , Medical Education in Western India: Grant Medical College and Sir Jamsetjee Jejeebhoy’s Hospital (Newcastle upon Tyne: Cambridge Scholars Publishing, 2019).

47 BL, IOR/P/278/5, May 1826, 1321.

48 BL, IOR/P/278/5, May 1826, 1324.

49 BL, IOR/P/278/5, May 1826, 1333–4.

50 BL, IOR/P/278/5, May 1826, 1325.

51 BL, IOR/P/278/5, May 1826, 1328–9.

52 BL, IOR/P/278/5, May 1826, 1339–53.

53 Pandya, *op. cit.* (note 31), 48–9.

54 ‘Liberality of the Indian Government’, *op. cit.* (note 40), 17–25.

55 BL, IOR/P/31/41, 30 December 1825, 147.

56 ‘Liberality of the Indian Government’, *op. cit.* (note 40), 24–5.

57 ‘Debates at the East India House’, *The Oriental Herald*, X, July-September 1826, 188.

58 BL, IOR/P/31/44, 13 January 1826, 260.

59 Pandya, *op. cit.* (note 31), 50.

60 BL, IOR/R/15/1/52, 21 December 1829, 100–1.

61 BL, IOR/F/4/1357/54154, 8 June 1832, 48–9.

62 Arnold, *op. cit.* (note 23), 54–8; Arnold, *op. cit.* (note 24), 62; Khaleeli, *op. cit.* (note 14), 87.

63 BL, IOR/F/4/1527/60296, 13 March 1834, 14–5.

64

William Knighton , The Private Life of an Eastern King (London: Hope and Co., 1855), 330 . See also: Katharine Bartsch and Peter Scriver , ‘The house of stars: Astronomy and the architecture of new science in early modern Lucknow (1831-49),’ in Samer Akkach (ed.), Ilm: Science, Religion and Art in Islam (Adelaide: University of Adelaide Press, 2019), 59–78 .

65 Iqbal Husain, ‘Awadh on the Eve of 1857 – Evidence of the Urdu Newspaper, *Tilism*,’ *Proceedings of the Indian History Congress*, 59 (1998), 765.

66 Rénald Lessard, ‘Le livre médical au sein du corps de santé canadien aux XVII^e^ et XVIII^e^ siècles’, *Canadian Bulletin of Medical History/Bulletin canadien d’histoire de la médecine*, 12, 2 (1995).

67

Christopher M. Parsons , A Not-So-New World. Empire and Environment in French Colonial North America (Philadelphia: University of Pennsylvania Press, 2018), 75–7.

68 Rare examples of publications that trace indigenous health practices in Canada or their relationship to imperial medicine include: Travis Hay , Inventing the Thrifty Gene. The Science of Settler Colonialism (Winnipeg: University of Manitoba Press 2021); James Burgess Waldram, D. Ann Herring and T. Kue Young , Aboriginal Health in Canada: Historical, Cultural and Epidemiological Perspectives (Toronto: University of Toronto Press, 2006); Maureen K. Lux , Medicine that Walks: Disease, Medicine, and Canadian Plains Native People, 1880-1940 (Toronto: University of Toronto Press 2001); Mary-Ellen Kelm , Colonizing Bodies. Aboriginal Health and Healing in British Columbia, 1900-50 (Vancouver: UBC Press, 1999); R. Geddes Large , Drums and Scalpels: From Native Healers to Physicians on the North Pacific Coast (Vancouver: Mitchell Press, 1968).

69

Dexnell Peters and Farid F. Youssef , ‘Historical perspectives on medical professionalism in the Caribbean’, West Indian Medical Journal (2017), DOI:10.7727/wimj.2017.106, 7–8; Juanita Barros , ‘Imperial connections and Caribbean medicine, 1900-1938’, in Laurence Monnais and David Wright (eds.), Doctors Beyond Borders: The Transnational Migration of Physicians in the Twentieth Century (Toronto: University of Toronto Press, 2016), 23 . See Willoughby’s article in this issue on the racial dynamics in medical education in the United States.

70

Richard W. Vaudry , Andrew Fernando Holmes. Protestantism, Medicine, and Science in Nineteenth-Century Montreal (Toronto: University of Toronto Press, 2020), 173 ; Jacques Bernier , ‘François Blanchet et le mouvement réformiste en médecine au début du XIX^e^ siècle’, Revue d’histoire de l’Amérique française, 34, 2 (1980), 226 11635704.

71 The British ‘Murder Act’, passed in London in 1752 and loosely applied in Canada until 1839, allowed judges to sentence people convicted of murder not only to the gallows but also to have their bodies dissected by anatomists. This act provided the legal framework for human dissection in several British colonies, see: ‘An Act for Better Preventing the Horrid Crime of Murder’, *Statutes of Great Britain* (1751), c. 37; Clare Anderson, ‘Execution and its aftermath in the nineteenth-century British Empire’, in Richard Ward (ed.), *A Global History of Execution and the Criminal Corpse* (Basingstokes: Palgrave Macmillan, 2015), 186–8. From the 1820s, human dissection in Canada was also often associated with body snatching from graves: Donald G. Lawrence , ‘“Resurrection” and Legislation or Body-Snatching in Relation to the Anatomy Act in the Province of Quebec,’ Bulletin of the History of Medicine, 32, 5 (1958), 408–2413584940 .

72 Measures to prevent scurvy were in place in Canada but as a general policy of the British navy: C. Stuart Houston , ‘Scurvy and Canadian Exploration,’ Canadian Bulletin of Medical History/Bulletin canadien d’histoire de la médecine, 7, 2 (1990), 161–710.3138/cbmh.7.2.16111622549.

73

John MacGregor , Observations on Emigration to British America (London: Longman, Rees, Orme, Brown and Green, 1829), 34 .

74 Parsons, *op. cit.* (note 67), 20.

75 Gilles Janson, ‘James Fisher,’ in *Dictionary of Canadian Biography* (subsequently DCB), http://www.biographi.ca/en/bio/fisher_james_6E.html, accessed 14 June 2023; Gilles Janson, ‘Charles Blake,’ in *DCB*, http://www.biographi.ca/en/bio/blake_charles_5E.html, accessed 14 June 2023.

76 Library and Archives Canada (subsequently LAC), MG11-CO42, vol. 50, reel C-11895, 525–6.

77
*Ibid.*, 534–54. See also: Rénald Lessard , ‘
*Directions pour la guérison du Mal de la Baie s^t^ Paul*: La première publication médicale canadienne,’ Canadian Bulletin of Medical History/Bulletin canadien d’histoire de la médecine, 12, 2 (1995), 369–7210.3138/cbmh.12.2.36911609085.

78 Rénald Lessard, ‘Les soins de santé au Canada aux XVII^e^ et XVIII^e^ siècles’, in Normand Séguin (ed.), *Atlas historique du Québec – L’institution médicale* (Quebec City: Presses de l’Université Laval, 1998), 25; Rénald Lessard, *Au temps de la petite vérole. La médecine au Canada aux XVII^e^ et XVIII^e^ siècles* (Quebec City: Septentrion, 2012), 346–7.

79

Sylvio Leblond , ‘La médecine dans la province de Québec avant 1847’, Les Cahiers des dix, 35 (1970), 73–5.

80

S. E. D. Shortt , ‘Physicians, science, and status: Issues in the professionalization of Anglo-American medicine in the nineteenth century’, Medical History, 27, 1 (1983), 53 10.1017/s0025727300042265PMC11392646339842; Rainer Baehre , ‘The Medical Profession in Upper Canada Reconsidered: Politics, Medical Reform, and Law in a Colonial Society,’ Canadian Bulletin of Medical History/Bulletin canadien d’histoire de la médecine, 12, 1 (1995), 103 10.3138/cbmh.12.1.10111609091; Jacques Bernier , La médecine au Québec. Naissance et évolution d’une profession (Quebec City: Presses de l’Université Laval, 1990); Denis Goulet, *Histoire du Collège des médecins du Québec, 1847-2022* (Quebec City: Septentrion, 2022).

81

Charles-Marie Boissonnault , Histoire de la Faculté de médecine de Laval (Quebec City: Presses de l’Université Laval, 1953), 88–98 ; Rénald Lessard , ‘Un emprunt éphémère: le Dispensaire de Québec, 1818-1820’, Cap-aux-Diamants, 4, 3 (1988), 56 ; Rénald Lessard, ‘Charles-Norbert Perreault,’ in *DCB*, http://www.biographi.ca/fr/bio/perrault_charles_norbert_6F.html, accessed 14 June 2023; Lewis Hertzman, ‘Anthony von Iffland,’ in *DCB*, http://www.biographi.ca/fr/bio/iffland_anthony_von_10F.html, accessed 14 June 2023.

82 Claude Galarneau, ‘L’enseignement médical à Québec (1800-1848)’, *Les Cahiers des dix*, 53 (1999), 42.

83

Marian A. Patterson , ‘The Life and Times of the Hon. John Rolph, M.D. (1793-1870)’, Medical History, 5, 1 (1961), 25–610.1017/s0025727300025898PMC103458213733371. The first source regarding this project dates from c. 1824: LAC, MG24-I121.

84

Brian Young , Patrician Families and the Making of Quebec: The Taschereaus and McCords (Montreal: McGill-Queen’s University Press, 2014), 204 .

85 Vaudry, *op. cit.* (note 70), 102.

86 While in Montreal, Sleigh became close to the city’s leading Irish businessmen such as Thomas McCord: William Willcocks Sleigh, *Statement by W. W. Sleigh, Esq.* (London: G. Odell, 1833); Harry Kuntz, ‘Science Culture in English-Speaking Montreal, 1815-1842’ (unpublished PhD thesis: Concordia University, 2010), 129 and 178; ‘Schola Medicinae’. *Quebec Mercury*, August 6, 1822, 251.

87

Joseph Hanaway and Richard Cruess , McGill Medicine, 1: The First Half Century, 1829-1885 (Montreal: McGill-Queen’s University Press, 1996), 11–3; Vaudry, *op. cit.* (note 70), 86–97; ‘Institution Medicale’. *Canadian Spectator*, March 12, 1823.

88 Vaudry, *op. cit.* (note 70), 81–2; K. David Milobar , ‘Château Clique’, in Gerald Hallowell (ed.), The Oxford Companion to Canadian History (Oxford: Oxford University Press, 2004), 124 .

89 Joseph Hanaway and John H. Burgess (eds.), *The General: A History of the Montreal General Hospital* (Montreal: McGill-Queen’s University Press, 2016), 15–6; Vaudry, *op. cit.* (note 70), 84–5. See also: Elizabeth Ann Kerr McDougall, ‘SOMERVILLE, JAMES (1775-1837),’ in *DCB*, http://www.biographi.ca/en/bio/somerville_james_1775_1837_7E.html, accessed 10 February 2023.

90 Hanaway and Cruess, *op. cit.* (note 87), 6, 10–4 and 145. See also: Hanaway and Burgess, *op. cit.* (note 89), 15–29.

91 Vaudry, *op. cit.* (note 70), 86–7.

92 ‘An Act for the Establishment of Free Schools and the Advancement of Learning in this Province,’ *Legislative Assembly and Council of the Province of Lower Canada*, 1801, c. 17.

93 McGill University Archives (subsequently MUA), RG 4, c. 302.

94 McGill University obtained the right to confer degrees in 1832, Vaudry, *op. cit.* (note 70), 99.

95 Vaudry, *op. cit.* (note 70), 73–8.

96 Bureau medical de Montreal. *La Minerve*, 4 July 1833.

97 ‘Governors’ of McGill College Minute Book’, MUA, 0004/0003/09003, 1829, 1.

98 Monica Marston, ‘Mountain, George Jehoshaphat,’ in *DCB*, http://www.biographi.ca/en/bio/mountain_george_jehoshaphat_9E.html, accessed 8 April 2021.

99 Baehre, *op. cit.* (note 80), 106.

100 C. K. Clarke, *A History of the Toronto General Hospital* (Toronto: William Briggs, 1913), 16–39.

101 G. M. Craig, ‘Strachan, John’, in *DCB*, http://www.biographi.ca/en/bio/strachan_john_9E.html, accessed 8 April 2021; David Mills, ‘Family Compact’, *The Canadian Encyclopedia*, https://www.thecanadianencyclopedia.ca/en/article/family-compact, accessed 8 April 2021.

102 Baehre, *op. cit.* (note 80), 106 and 112–3.

103

Martin L. Friedland , The University of Toronto: A History (Toronto: University of Toronto Press, 2002), 3 and 17–9; William F. A. Boys , ‘Early Days of the University’, The University of Toronto Monthly, 2, 3 (1901), 3–36 .

104 ‘King’s College. Faculty of Medicine, Syllabus for the winter session, 1844’, University of Toronto Archives, A1973-0022/003 (12); Barrie Dyster, ‘Gwynne, William Charles,’ in *DCB*, http://www.biographi.ca/en/bio/gwynne_william_charles_10E.html, accessed 8 April 2021.

105 National Archives of India, Lawspet Records Centre, no. 116, ‘Copies de lettres du docteur Bourdier et divers regardant la médecine au XVIII^e^ siècle à Pondichéry, 1764-1787,’ 103–25; National Archives of India, Lawspet Records Centre, no. 348, ‘Trauttmans Dorff (La Comptesse [sic]). Catalogue de la cargaison du vaisseau impérial et pièces, 1789-1791,’ 143–4.

106

Thomas Neville Bonner , Becoming a Physician. Medical Education in Britain, France, Germany, and the United States (Baltimore and London: John Hopkins University Press, 2000), 169–70.

107 Peter Burroughs, ‘Ramsay, George, 9^th^ Earl of Dalhousie,’ in *DCB*, http://www.biographi.ca/en/bio/ramsay_george_7E.html, accessed 10 June 2023; Joseph Workman, ‘Medical Inaugural Dissertation on Asiatic Cholera’ (MD thesis: McGill University, 1835).

108 Arnold, *op. cit.* (note 24), 62–3.

109 Alavi, *op. cit.* (note 13), 880.

110 Harrison, *op. cit.* (note 25), 96–7.

111 Arnold, *op. cit.* (note 24), 64.

112 Sen and Das, *op. cit.* (note 42), 486. Madhusudan Gupta had previously worked at the NMI in Calcutta, including as a translator: Bhattacharya, *op. cit.* (note 34), 244.

113

Jayanta Bhattacharya , ‘The First Dissection Controversy: Introduction to Anatomical Education in Bengal and British India’, Current Science, 101, 9 (2011), 1228 .

114 Harrison, *op. cit.* (note 24), 185–7. See also: Bhattacharya, *op. cit.* (note 34), 253. The source on the number of bodies dissected is: Allan Webb, *Pathologia Indica, or the Anatomy of Indian Diseases* (Calcutta, Thacker and Co., 1848), 237.

115 Alavi, *op. cit.* (note 13), 896.

116 Arnold, *op. cit.* (note 23), 246–54; Arnold, *op. cit.* (note 24), 70.

117 ‘An Act to Establish Boards of Health within this Province, and to enforce an Effectual System of Quarantine’, *Provincial Statutes of Lower Canada*, 1832, c. 16.

118 Marcel Rheault and Georges Aubin, *Médecins et patriotes. 1837-1838* (Quebec: Septentrion, 2006).

119

John George Lambton , Report on the Affairs of British North America from the Earl of Durham (London: House of Commons of the United Kingdom, 1839).

120 ‘Acte pour incorporer l’École de Médecine de Québec’, *Statuts provinciaux du Canada*, 1845, c. 80; ‘Acte pour incorporer l’École de Médecine et de Chirurgie de Montréal’, *Statuts provinciaux du Canada*, 1845, c. 81.

121 In 2013, doctors graduating in India accounted for approximately 10% of all registered doctors in the United Kingdom: Leila Mehdizadeh et al, ‘Prevalence of GMC performance assessments in the United Kingdom: A retrospective cohort analysis by country of medical qualification,’ The BMC Medical Education, 17, 67 (2017),28372544 10.1186/s12909-017-0903-6PMC5379692 Table 4.

